# Enhancing Nursing Competencies: An Assessment of Knowledge and Attitudes Toward Dental Trauma Management Among Nursing Students—An Interventional Study

**DOI:** 10.3390/nursrep14040275

**Published:** 2024-11-29

**Authors:** Beatriz Emilia Carrion-Ruiz, Alberto Cabrera-Fernandez, Isabel Crespo-Gallardo, Daniel Cabanillas-Balsera, Juan J. Segura-Egea, Manuel Pabón-Carrasco, Jenifer Martin-Gonzalez

**Affiliations:** 1 Department of Stomatology, Faculty of Dentistry, University of Seville, 41009 Sevilla, Spain; beacarrui@alum.us.es (B.E.C.-R.); acabrera5@us.es (A.C.-F.); icrespo@us.es (I.C.-G.); segurajj@us.es (J.J.S.-E.); 2 Faculty of Nursing, Physiotherapy and Podiatry, University of Seville, 41009 Sevilla, Spain; 3 Research Group PAIDI-CTS-1050: 7 “Complex Care, Chronicity and Health Outcomes”, University of Seville, 41009 Sevilla, Spain

**Keywords:** nursing, knowledge, health education, dental education, tooth injuries, first aid, dental trauma, traumatology, emergency care, allied dental personnel

## Abstract

Introduction: Traumatic dental injuries (TDIs) present a significant challenge for healthcare professionals. Nurses, often the first point of contact for patients, may lack essential knowledge in dental trauma first aid, as noted in the existing literature. Objective: To assess the knowledge of traumatic dental injuries (TDIs) among undergraduate nursing students before and after a targeted educational intervention. Materials and Methods: This quasi-experimental study evaluated the effectiveness of an educational intervention involving 300 nursing students from two universities in Seville. The educational intervention was led by specialist dentists. A pre-test survey was administered to assess students’ baseline knowledge. The session included a lecture on TDI management, followed by a simulation in which students practiced emergency splinting techniques for referral to a dentist. A post-test survey was then conducted to measure changes in students’ knowledge and attitudes towards dental trauma. Results: Only 25.4% of students had prior training in dental trauma. After the intervention, there was a significant improvement in students’ self-assessed knowledge of first aid for TDIs (*p* < 0.05). A high percentage of students also reported increased confidence in their ability to reimplant a tooth. Statistical analysis of pre- and post-intervention survey results showed a substantial increase in average scores (*p* < 0.05). Conclusions: Nursing students initially exhibited limited knowledge in managing dental trauma but demonstrated a positive attitude toward learning this new skill. The targeted educational intervention significantly enhanced their understanding, underscoring the need to incorporate such training into nursing curricula. By improving nursing students’ competence in managing dental injuries, this training can help better preserve dental structures and improve the prognosis for dental trauma cases.

## 1. Introduction

In recent years, the prevalence of traumatic dental injuries (TDIs) has increased significantly [1,2]. Despite the oral cavity representing only 1% of the body, TDIs account for 5% of all bodily injuries [3,4]. According to a recently published systematic review [5], the prevalence of dental trauma is approximately 15%, with similar findings reported in Spain [6,7]. The avulsion of a permanent tooth accounts for 0.5–3% of TDIs [8].

The prevalence of TDI varies by age, with the highest incidence occurring between the ages of 7 and 12, during school age [9,10,11]. The upper incisors are the most commonly affected teeth [9,12] due to their prominent position in the mouth. Several predisposing factors for TDIs have been identified in the literature, including protruding upper teeth, overjet (greater than 3 mm), anterior open bite, class II malocclusion, incompetent lips, and mouth breathing [13,14,15,16,17], with increased overjet being one of the primary risk factors [18]. Additionally, the rise of new technologies introduced additional risk factors, particularly the accidental dropping of smartphones onto the face during periods of rest periods. This is linked to the weight of these devices and their widespread use globally [19,20].

The etiology of TDIs is closely linked to age, with different causes and patterns observed in preschoolers, school-age children, and adults. Common causes of TDIs include traffic accidents, sports injuries, assaults, and falls [21,22,23].

One of the most frequent TDIs is dental avulsion, which involves the complete displacement of a tooth from its socket. Avulsion accounts for 0.5% to 16% of all dental traumas, with the highest incidence for avulsion of permanent anterior teeth occurring between the ages of 7 and 9 years. Several studies have shown that the survival rate of an avulsed permanent tooth significantly improves if first aid and treatment are provided within the first 15 min following the injury [24]. A key factor in the successful reimplantation of a permanent tooth is the preservation of the periodontal ligament on the root. To prevent dehydration of the avulsed tooth, it must be stored in an appropriate medium. Therefore, timely and proper knowledge of emergency management is essential to ensure a favorable prognosis for the tooth [25].

The improper initial management of dental trauma can significantly impact the viability of the affected tooth and lead to psycho-emotional issues for the patient, stemming from both functional and aesthetic consequences. This, in turn, can negatively affect the patient’s overall quality of life [26,27,28]. While general dental practitioners typically have a moderate understanding of TDI management, specialists such as endodontists and pediatric dentists are more adept at handling these injuries due to their specialized knowledge in dental traumatology [29]. Moreover, medical doctors often lack sufficient expertise in managing dental trauma [30,31]. Therefore, prompt intervention by trained professionals in the initial management of TDI can significantly improve the prognosis of the affected tooth.

Some studies emphasize the importance of increasing awareness among all healthcare providers who may be present at the scene of an accident or in the immediate aftermath, including those on the first line of response [32]. Coaches, recreational leaders, and first responders should all know the initial management of an avulsed tooth. Additionally, patient and parental education, along with timely medical care, can enhance treatment outcomes. The use of protective mouthguards in high-risk contact sports is also recommended as a preventive measure to reduce the incidence of dental injuries [16].

It is important to highlight that dental injuries are more common in children between the ages of 7 and 12, making it essential that those responsible for their care, such as parents, primary school teachers, and school nurses, are informed about how to manage these injuries. Nurses play a crucial role in emergency preparedness and are often the only healthcare professionals available to handle dental emergencies. Training nurses in this area is logical, as their emergency training makes them potential allies of dentists. Furthermore, many dental avulsions occur at night, when most dental clinics are closed, making hospital emergency departments the primary point of care for such traumas. In both school and hospital settings, nurses play a pivotal role in preserving dental integrity [33,34].

Several studies have also assessed the knowledge levels of various professionals who may serve as first responders, such as teachers, parents, or sports coaches, revealing an insufficient level of knowledge in this area [35,36,37].

It is crucial to emphasize the importance of healthcare professionals in managing such emergencies. They must possess the necessary qualifications to properly address TDI emergencies, based on scientific evidence [38]. However, the available literature indicates a deficient level of knowledge among non-dental healthcare professionals, particularly in nursing, regarding the initial management of TDI [36,38,39,40,41].

Taking into account the widespread lack of knowledge about first aid procedures in cases of dental trauma and the importance of correctly addressing TDIs, this study is particularly justified due to the significant knowledge gap in the field of nursing regarding TDIs [42,43]. The aim of the present study was to assess the knowledge and attitudes toward dental trauma management among nursing students, evaluating the impact of a training workshop on the management of dental avulsion.

The alternative hypothesis suggests that there is a positive inclination toward gaining new competencies in first aid, particularly in managing dental trauma. Additionally, educational interventions that combine lectures with simulation have proven effective in improving the acquisition of these skills.

## 2. Materials and Methods

### 2.1. Study Design and Ethics

A quasi-experimental pre-test–post-test study was designed to evaluate the knowledge and attitudes of nursing students regarding the management of dental trauma by surveys. A total of 300 nursing students were recruited from the Red Cross School of Nursing (Seville) and the University School of Osuna (University of Seville) over three academic years (2022–2023, 2023–2024, and 2024–2025). Accordingly, a multicenter, non-randomized experimental study was conducted, with pre- and post-intervention assessments carried out via a survey following an educational intervention.

The study was approved by the ethics committee (code *1485-N-23*). The MINORS tool (Methodological Index for Non-Randomized Studies) was used to evaluate the methodological quality of non-randomized studies [44] (Table S1).

### 2.2. Criteria

The inclusion criteria required participants to be enrolled in a nursing degree program at a Spanish university and to have completed at least 75% of their studies (fourth year). In addition, it was verified that all participants had completed courses related to first aid, primary care, or trauma management, to ensure homogeneity in the sample regarding prior competencies. All nursing students who were not in the last year of their degree or who did not have courses related to first aid, primary care, or trauma management were excluded. Nursing students who had completed a degree in dentistry or maxillofacial medical specialty were also excluded. During the study, there were no participant dropouts as the post-test data collection was conducted immediately. Additionally, 100% of the students contacted provided consent to participate, likely because the educational intervention was not part of any academic evaluation and had no impact on their grades. Students were motivated by the opportunity to acquire new practical skills, and the professors leading the sessions were external to the institutions where the students were enrolled, which helped mitigate any perception of coercion.

### 2.3. Questionnaire

A descriptive questionnaire consisting of 20 closed-end questions was used for data collection. These questions were based on surveys from previous studies [36] and were reviewed by researchers and professors from the Postgraduate Program in Clinical Endodontics at the University of Seville. The questionnaire was divided into four sections: sociodemographic data, previous training on the subject, attitudes towards the topic, and 11 questions related to knowledge on dental trauma management (Table S2).

**Figure 1:**
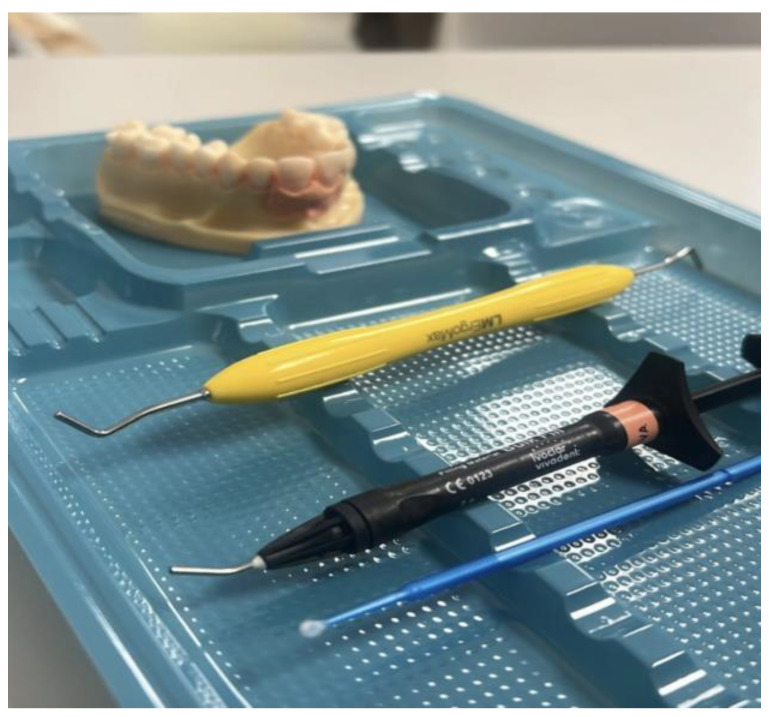
Anatomical model and instruments during practical session.

Following the initial evaluation using the questionnaire, a training workshop was conducted. Two licensed dentists with a master’s degree in Endodontics and over 10 years of professional experience were previously trained to deliver the workshop. The workshop consisted of two sections: a 40 min theoretical session, followed by a 30 min practical session using an anatomical model simulating a dental avulsion case (Figure 1). A total of eight sessions were held between 2022 and 2024.

Data were collected digitally, with students completing anonymous online questionnaires using their smartphones. All surveys were evaluated by two endodontics specialists, who assigned scores ranging from 0 to 11 based on the correctness of the responses. Each correct answer was awarded one point, with a maximum score of eleven points.

### 2.4. Statistical Analysis

The sample size was determined to ensure a statistical power of 0.95, with an alpha level of 0.05 and a medium effect size (calculated using the Chi-square test method, G*Power 3.0.10, Franz Faul, University of Kiel, Germany). A total of 298 participants were required [45].

Data analysis was performed using SPSS Statistics Version 29.0 (IBM Corporation; Armonk, NY, USA). Initially, a descriptive analysis was conducted using frequency tables, providing a numerical representation in percentages based on the total number of responses for each question. An inferential analysis followed, using the Chi-square test to determine whether significant differences existed between pre-intervention and post-intervention results. Additionally, a paired sample *t*-test was performed to analyze the scores obtained by the pre- and post-intervention groups. Statistical significance was set at *p* < 0.05.

## 3. Results

Eighty-six percent (257 participants) were females, while 14.3% (43 participants) were males. Nineteen participants (6.3%) had previous experience in the dental field, specifying that 5% of participants held a degree in dental hygiene or were a clinical assistant (Table 1).

In response to the previous questionnaire, 45.3% (136 participants) were able to distinguish between primary and permanent teeth. Additionally, 90% (270 participants) indicated they would attempt to preserve the tooth fragment, and 81.7% (196 participants) stated they would keep it in a moist environment. Regarding the clinical scenario of an avulsed tooth, 68.3% (205 participants) believed the tooth could be re-implanted, while 23.3% (70 participants) felt capable of performing the re-implantation themselves. When asked about the proper management of an avulsed tooth, 48.3% (145 participants) reported they would rinse the tooth under running water for 10 s. Finally, 82% (245 participants) confirmed they would refer the patient to a dentist (Table 2).

The analysis by center revealed statistically significant differences in the pre-test assessment of seven knowledge items between nursing schools. These differences may be attributed to the fact that students at the Red Cross Centre had more prior experience with avulsion (76.3% (n = 58) vs. 23.6% (n = 18)) and previous training in dental traumatology (69.2% (n = 47) vs. 30.8% (n = 21)). However, no significant differences were observed between the centers after the educational intervention (Table 3).

Following the intervention, there was a notable improvement in the rate of correct answers across all questions assessing knowledge of first aid in dental trauma. The mean scores increased from 5.85 ± 1.91 to 10.35 ± 0.95 (Table 3).

## 4. Discussion

The results indicate an inadequate level of knowledge regarding the proper management of traumatic dental emergencies, although students show a proactive attitude toward acquiring these competencies. Educational intervention demonstrated its ability to reverse this situation, regardless of the educational institution involved. Moreover, prior experience proves to be an asset in acquiring new skills, as it facilitates the better internalization of information. This highlights the value of incorporating clinical cases, as well as both low- and high-fidelity simulators, into educational programs.

Currently, no specific training in dental trauma is included in the nursing degree curriculum at Spanish universities, as it is not considered a core or specialized competency of nursing graduates. School nurses, as the first assistance available in trauma situations with school pupils, should have proper knowledge of the emergency early management of traumatic dental injuries to ensure the best possible prognosis for the injured teeth. Studies from different countries around the world demonstrated that school nurses lack proper knowledge of dental injury management and that a pressing need for training and education exists. This also applies to school teachers, another professional group whose knowledge and attitudes toward dental trauma have been assessed. [39,46,47,48,49,50,51,52,53,54].

It is noteworthy that 22.5% of respondents reported witnessing dental trauma—a figure lower than that found in similar studies among nurse practitioners [40]. This suggests that professional practice may increase exposure to such events, emphasizing the importance of prior knowledge before entering the workforce.

One of the most critical aspects of properly managing a traumatic dental injury is the correct identification of a permanent tooth, distinguishing it from a temporary one, and vice versa. While only 45.4% of respondents considered themselves capable of making this distinction in the pre-intervention phase, this percentage significantly increased to 99.1% following the training session.

Dental avulsion is one of the most serious injuries where immediate repositioning can determine the tooth’s prognosis [24]. This justifies the emphasis placed on avulsion-related questions in our questionnaire, as well as a significant part of the intervention. The goal is not to enable nurses to perform tasks exclusively to dental professionals but rather to ensure that they can manage emergencies, preserving the tooth in an optimal condition until a specialist can assess the extent of the injury.

Initially, students were asked about the viability of repositioning an avulsed tooth after the accident. In the pre-intervention questionnaire, 68.3% believed it was possible, and this percentage significantly increased to 94.8% post-intervention. These results contrast with those of Hugar, S.M. and colleagues, where 41% of nurses reported being unaware that a tooth could be replanted in the initial assessment [33].

When asked about their willingness to reposition an avulsed tooth, only 23.3% expressed willingness to do so initially, but this increased significantly to 92.6% following the training. This increase is highly significant, given the importance of timely repositioning for a favorable prognosis. Delays beyond the first 15 min can lead to irreversible damage to the periodontal ligament cells, reducing the chances of successful treatment [10,22]. More pessimistic initial percentages have been observed in analogous studies from other countries [41].

If immediate reimplantation is not possible, and delayed reimplantation by a dental professional is opted for, the storage medium in which the tooth is kept becomes crucial, as it determines the survival of periodontal ligament cells. Prolonged exposure to a dry environment of 20–30 min can cause the loss of normal metabolism and physiology in these essential cells for tooth reattachment [24]. Prior to the intervention, 64.6% of respondents correctly identified the appropriate transport medium, and this percentage increased to 99.6% afterwards. Although there is a significant difference, the initial 64.6% reflects a reasonable level of knowledge, potentially due to the students’ familiarity with the cytotoxic effects of antiseptics commonly used in healthcare.

Not all liquid mediums equally preserve cellular physiology, and although no medium is 100% compatible with the survival of periodontal ligament cells, milk is considered the “gold standard” by the International Association of Dental Traumatology [9] due to its high compatibility [55]. In the initial phase of the study, when students were asked about the type of liquid medium, 25% selected fresh milk—a figure that rose to 93.5% following the educational intervention.

Additionally, to quantify the improvement in participants’ knowledge of first aid in dental trauma, the pre- and post-intervention questionnaires were scored based on questions that could be evaluated as either correct or incorrect. A statistically significant difference was observed when comparing the pre- and post-intervention scores, consistent with the findings of Al Sari, S. [56].

Our intervention was carried out in a single session, although it included an initial theoretical part, with real clinical images, followed by a practical workshop. The results of intervention studies in other populations, such as primary school teachers [57], suggest that a single session is insufficient and that information needs to be reinforced over time.

Some authors argue that smartphone applications may be more effective than lectures delivered by professionals [58]. A poster entitled “Save your teeth” has been recommended as an educational tool in schools, and teachers who have seen the poster responded significantly better when compared with colleagues who had no previous exposure to the poster’s information [59].

A notable strength of the current study is the inclusion of real clinical simulation using anatomical models during the intervention, making it the only study to date to incorporate such an activity. However, the study has certain limitations, including the lack of follow-up to assess the long-term impact of the training. Additionally, a national sample would be needed to determine whether the knowledge deficits observed are consistent across Spain, as recent studies indicate a lack of research in this area across Europe [60]. Another limitation is the lack of a control group to evaluate the intervention’s impact, as is common in quasi-experimental studies; however, this could be a potential future line of research once this competency is established in nursing programs. A comparison between different universities where this approach is implemented could help assess how the increase in skills translates into healthcare practice.

This study has significant clinical implications, reinforcing the role of school nurses as key healthcare providers in educational settings. It also underscores the importance of multidisciplinary collaboration to enhance patient care. Training for both nurses and general practitioners serves a common goal: ensuring the proper preservation of dental structures and their subsequent referral to dental specialists for further assessment.

A survey of nurses found that 30% of respondents encounter children with dental trauma on a daily or weekly basis. However, only 13% received training on traumatic dental injuries (TDIs) during their undergraduate studies, and 32% reported having received no training at all [42]. This highlights a significant gap in dental education within undergraduate medical programs, with a particular lack of instruction on dental trauma despite its frequent occurrence in medical settings. The study’s responders suggested that they would benefit from online resources and regular training on pediatric TDIs, as well as an easy-to-use decision-making tool to guide families [42]. Improving dental trauma education for all emergency department healthcare professionals is crucial to boosting their confidence and equipping them with the skills to triage and manage patients effectively. Furthermore, directing families to appropriate services could improve both outcomes and the overall experience for children affected by traumatic dental injuries [42]. Given the frequency of such emergencies, it is imperative that nurses possess adequate knowledge in the management of traumatic dental injuries [43].

The results of the present study highlight the potential for enhanced collaboration between dentists and nurses. Collaborative leadership is where a mutually beneficial relationship is established between two or more parties, who work towards common goals by sharing authority, responsibility and accountability for achieving results (this is the goal) [61]. Consequently, the alternative hypotheses, which assumed a knowledge deficit in the assessed area and a proactive attitude towards learning this competency, are confirmed. Additionally, the value of educational interventions led by other disciplines, alongside simulation as an effective learning tool, is reinforced. Collaborative efforts could include dentists contributing to postgraduate training for nurses through workshops, seminars, and continuing professional development programs, or developing resources and protocols for managing dental trauma that nurses and allied professionals can use in emergency situations (e.g., instructional videos provided by professional associations or accredited dentists). Another possibility is the establishment of an emergency advisory hotline run by a team of dentists, specifically aimed at guiding nurses and allied professionals in cases of dental trauma and emergencies. From this initiative, telemedicine and tele-dentistry services could further support professionals and patients. Such collaborative efforts align with the new Spanish University Law (LOSU), which promotes interdisciplinary cooperation in healthcare education and practice [62].

## 5. Conclusions

In conclusion, nursing students exhibited limited knowledge of managing dental trauma emergencies. However, the implementation of an educational intervention led to a significant improvement in their understanding of the appropriate management of these injuries. Furthermore, students demonstrated a proactive attitude toward acquiring the necessary competencies to improve the quality of care they provide. This proactive approach includes a greater willingness to collaborate with other disciplines, which is crucial for delivering comprehensive and effective care in dental emergency situations.

## Figures and Tables

**Table 1:** Socio-demographic variables and previous education of participants.

Socio-Demographic Variables	N = 300	Frequency (n)	Percent (%)
**Sex**	Men	43	14.3
	Women	257	85.7
**Centre**			
	Red Cross Centre	163	54.2
	University School of Osuna	137	45.8
**Previous training**			
**Previous dental experience**	Yes	19	6.3
	No	281	93.8
**Previous dental trauma training**	Yes No	76 224	25.4 74.6
**Previous avulsion experience**	Yes No	68 232	22.5 77.5
	**Mean (SD)**	**95% IC**	**Max/Min**
**Age (years)**	22.28(3.21)	21.93–22.81	45/19

Notes: 95% IC = 95% Confidence Interval; Max/Min = Maximum/Minimum; N = Total sample size; n = Absolute frequency; SD = Standard Deviation.

**Table 2:** Inferential analysis of the correct response rate between the pre- and post-educational intervention groups.

ASK	PRE GROUP N = 300	POST GROUP N = 300	STATISTICAL SIGNIFICANCE
**Self-concept** (Do you think you have the knowledge necessary to manage a traumatic dental injury?)	Yes 19.6% (n = 59) Not 80.4% (n = 241)	Yes 87.4% (n = 262) Not 12.6% (n = 38)	* *p* < 0.0001 Cramer’s V 0.679
**Predisposition** (Do you think you need more knowledge/training regarding dental traumatology?)	Yes 85.8% (n = 257) Not 14.2% (n = 43)	Yes 83.5% (n = 250) Not 16.5% (n = 50)	*p* = 0.479
**Distinction** (Are you able to distinguish be-tween a temporary tooth and a permanent one)	Yes 45.4% (n = 136) Not 54.6% (n = 164)	Yes 99.1% (n = 297) Not 0.9% (n = 3)	* *p* < 0.0001 Cramer’s V 0.596
**Case 1A** (Is this a temporary or perma-nent tooth?)	Temporary 17.1% (n = 51) Permanent 82.9% (n = 249)	Temporary 6.1% (n = 18) Permanent 93.9% (n = 282)	* *p* < 0.0001 Cramer’s V 0.171
**Case 1B** (Would you keep the frag-ments?)	Yes 90% (n = 270) Not 10% (n = 30)	Yes 98.3% (n = 295) No 1.7% (n = 5)	* *p* < 0.0001 Cramer’s V 0.174
**Case 1C** (What type of medium would you preserve it on?)	Wet 81.7% (n = 245) Dry 16.7% (n = 55)	Wet 99.6% (n = 299) Dry 0.4% (n = 1)	* *p* < 0.0001 Cramer’s V 0.304
**Case 2A** (Do you think it is possible to relocate the avulsed tooth?)	Yes 68.3% (n = 205) Not 31.7% (n = 95)	Yes 94.8% (284) Not 5.2% (16)	* *p* < 0.0001 Cramer’s V 0.339
**Case 2B** (Would you be able to reimplant it?)	Yes 23.3% (n = 71) Not 76.3% (n = 229)	Yes 92.6% (n = 278) Not 7.4% (n = 22)	* *p* < 0.0001 Cramer’s V 0.702
**Case 2C** (In what time limit do you think it should be relocated?)	Any time 2.1% (n = 6) First 5 h 14.2% (n = 43) After two hours to rehydrate 15.8% (n = 47) As soon as possible, without limit 48.8% (n = 146) In the first 24 h 19.2% (n = 58)	Any time 0.4% (n = 1) First 5 h 8.3% (n = 25) After two hours to rehydrate 3.9% (n = 12) As soon as possible, without limit 83.5% (n = 250) In the first 24 h 3.9% (n = 12)	* *p* < 0.0001 Cramer’s V 0.380
**Driving** (Given that the tooth is dirty, how would you handle it?)	I would replace without washing 0.8% (n = 2) Wash with saliva 3.8% (n = 11) Wash 10 sec. Under water 48.3% (n = 145) Wash with milk 8.3% (n = 25) Wash with soap 7.5% (n = 23) Rub with brush 31.3% (n = 94)	I would replace without washing 0.4% (n = 1) Wash with saliva 0.4% (n = 1) Wash 10 sec. Under water 82.2% (n = 247) Wash with milk 16.5% (n = 50) Wash with soap 0.4% (n = 1) Rub with brush 0% (n = 0)	* *p* < 0.0001 Cramer’s V 0.505
**Transport** (What type of transportation do you consider most appropriate?)	Liquid medium 64.6% (n = 194) Cold compress 15.8% (n = 47) Child’s mouth 4.6% (n = 14) Adult mouth 0.4% (n = 1) Wrapped in paper 5.4% (n = 16) Plastic container 9.2% (n = 28)	Liquid medium 94.3% (n = 283) Cold compress 1.3% (n = 4) Child’s mouth 0.9% (n = 3) Adult mouth 3.5% (n = 6) Wrapped in paper 0% (n = 0) Plastic container 0 (n = 0)	* *p* < 0.0001 Cramer’s V 0.430
**Medium type** (What type of medium do you consider most appropriate?)	Fresh water 15.8% (n = 47) Milk 25% (n = 75) Alcohol 1.7% (n = 5) Serum 36.7% (n = 110) Ice water 2.9% (n = 9) Antiseptic solution 17.9% (n = 54)	Fresh water 3.5% (n = 10) Milk 93.5% (n = 281) Alcohol 3% (n = 9) Serum 0% (n = 0) Ice water 0% (n = 0) Antiseptic solution 0% (n = 0)	* *p* < 0.0001 Cramer’s V 0.699
**Derivation** (Which professional do you con-sider to be your choice?)	Pediatrician 2.9% (n = 9) Dentist 81.7% (n = 245) Family doctor 0.8% (n = 2) Maxillofacial 14.6% (n = 44)	Pediatrician 0% (n = 0) Dentist 97.4% (n = 292) Family doctor 0% (n = 0) Maxillofacial 2.6% (n = 8)	* *p* < 0.0001 Cramer’s V 0.258
**Score mean ± SD** **IC 95%**	5.85 ± 1.99 5.59–6.10	10.35 ± 0.95 10.22–10.47	* *p* < 0.0001 Cohen’s d = 0.620

Notes: IC 95% = 95% confidence interval; N = Total sample size; n = Absolute frequency; Sec: Seconds; SD = standard deviation. Effect size = Cohen’s d = 0.2: Small effect size; 0.5: Moderate effect size; 0.8: Large effect size; Cramer’s V = 0–0.1: Very weak association; 0.1–0.3: Weak association; 0.3–0.5: Moderate association; 0.5–1: Strong association. Statistical significance = * *p* < 0.05.

**Table 3:** Inferential analysis of the rate of correct answers between the pre- and post-educational intervention groups taking into account the educational center.

ASK	PRE-GROUP		POST GROUP	
	**Cruz Roja** **n = 167**	**Osuna** **n = 137**	**Cruz Roja** **n = 167**	**Osuna** **n = 137**
**Self-concept** (Do you think you have the knowledge necessary to manage a traumatic dental injury?)	Yes 23.1% (n = 39) No 76.9% (n = 128)	Yes 15.5% [17] No 84.5% (n = 116)	Yes 89.7% (n = 150) No 10.3% (n = 17)	Yes 85.1% (n = 117) No 14.9% (n = 20)
Statistical significance	*p* = 0.138 Cramer’s V 0.096		*p* = 0.297 Cramer’s V 0.069	
**Predisposition** (Do you think you need more knowledge/training regarding dental traumatology?)	Yes 90% (n = 150) No 10% (n = 17)	Yes 80.9% (n = 111) No 19.1% (n = 26)	Yes 79.3% (n = 132) No 20.7% (n = 35)	Yes 87.7% (n = 120) No 12.3% (n = 17)
Statistical significance	*p* = 0.044 * Cramer’s V 0.130		*p* = 0.086 Cramer’s V 0.113	
**Distinction** (Are you able to distinguish between a temporary tooth and a permanent one)	Yes 55.4% (n = 93) No 44.6% (n = 74)	Yes 33.6% (n = 46) No 66.4% (n = 91)	Yes 99.1% (n = 166) No 0.9% (n = 1)	Yes 99.1%(n = 136) No 0.9% (n = 1)
Statistical significance	*p* < 0.001 *** Cramer’s V 0.218		*p* = 0.990 Cramer’s V 0.001	
**Case 1A** (Is this a temporary or permanent tooth?)	Temporary 15.4% (n = 26) Permanent 84.6% (n = 141)	Temporary 19.1% (n = 26) Permanent 80.9% (n = 111)	Temporary 7.8% (n = 13) Permanent 92.2% (n = 154)	Temporary 4.4% (n = 6) Permanent 95.6% (n = 131)
Statistical significance	*p* = 0.447 Cramer’s V 0.049		*p* = 0.285 Cramer’s V 0.071	
**Case 1B** (Would you keep the fragments?)	Yes 96.2% (n = 161) No 3.8% (n = 6)	Yes 82.7% (n = 113) No 17.3% (n = 24)	Yes 100% (n = 167) No 0% (n = 0)	Yes 96.5%(n = 132) No 3.5% (n = 5)
Statistical significance	*p* < 0.001 *** Cramer’s V 0.223		*p* = 0.052 Cramer’s V 0.134	
**Case 1C** (What type of medium would you preserve it on?)	Wet 80.8% (n = 135) Dry 19.2% (n = 32)	Wet 82.7% (n = 113) Dry 13.6% (n = 24)	Wet 100% (n = 167) Dry 0% (n = 0)	Wet 99.1%(n = 136) Dry 0.9% (n = 1)
Statistical significance	*p* = 0.053 Cramer’s V 0.156		*p* = 0.312 Cramer’s V 0.067	
**Case 2A** (Do you think it is possible to relocate the avulsed tooth?)	Yes 83.8%(n = 140) No 16.2% (n = 27)	Yes 50.4% (n = 69) No 49.6% (n = 58)	Yes 96.6% (n = 161) No 3.4% (n = 6)	Yes 93% (n = 127) No 7% (n = 10)
Statistical significance	*p* < 0.001 ***Cramer’s V 0.363		*p* = 0.224 Cramer’s V 0.080	
**Case 2B** (Would you be able to reimplant it?)	Yes 35.4% (n = 60) No 63.8% (n = 107)	Yes 9.1% (n = 12) No 90.9% (n = 125)	Yes 91.4% (n = 153) No 7.8% (n = 14)	Yes 93.9% (n = 129) No 43.8% (n = 8)
Statistical significance	*p* < 0.001 *** Cramer’s V 0.318		*p* = 0.539 Cramer’s V 0.073	
**Case 2C** (In what time limit do you think it should be relocated?)	Any time 3.1% (n = 5) First 5 h 8.5% (n = 14) After two hours to rehydrate 13.8% (n = 23) As soon as possible, without limit 57.7% (n = 96) In the first 24 h 16.9% (n = 28)	Any time 0.9% (n = 1) First 5 h 20.9% (n = 29) After two hours to rehydrate 18.2% (n = 25) As soon as possible, without limit 38.2% (n = 52) In the first 24 h 21.8% (n = 30)	Any time 0.9% (n = 1) First 5 h 4.3% (n = 7) After two hours to rehydrate 3.4% (n = 6) As soon as possible, without limit 88.8% (n = 149) In the first 24 h 2.6% (n = 4)	First 5 h 12.3% (n = 17) After two hours to rehydrate 4.4% (n = 6) As soon as possible, without limit 78.1% (n = 107) In the first 24 h 5.3% (n = 7)
Statistical significance	*p* < 0.001 *** Cramer’s V 0.241		*p* = 0.117 Cramer’s V 179	
**Driving** (Given that the tooth is dirty, how would you handle it?)	I would replace without washing 1.5% (n = 2) Wash with saliva 5.4% (n = 9) Wash 10 sec. Under water 50% (n = 84) Wash with milk 8.5% (n = 14) Wash with soap 5.4% (n = 9) Rub with brush 29.2% (n = 49)	Wash with saliva 1.8% (n = 2) Wash 10 sec. Under water 46.4% (n = 64) Wash with milk 8.2% (n = 11) Wash with soap 10% (n = 14) Rub with brush 33.6% (n = 46)	I would replace without washing 0.9% (n = 1) Wash with saliva 0.9% (n = 1) Wash 10 sec. Under water 75% (n = 126) Wash with milk 23.3% (n = 39)	Wash 10 sec. Under water 89.5% (n = 123) Wash with milk 9.6% (n = 13) Wash with soap 0.9% (n = 1)
Statistical significance	*p* = 0.312 Cramer’s V 0.157		*p* < 0.058 Cramer’s V 0.218	
**Transport** (What type of transportation do you consider most appropriate?)	Liquid medium 73.8% (n = 123) Cold compress 12.3% (n = 20) Child’s mouth 6.9% (n = 12) Adult mouth 0.8% (n = 1) Wrapped in paper 1.5% (n = 3) Plastic container 4.6% (n = 8)	Liquid medium 53.6% (n = 73) Cold compress 20% (n = 28) Child’s mouth 1.8% (n = 2) Wrapped in paper 10% (n = 14) Plastic container 14.5% (n = 20)	Liquid medium 94% (n = 158) Cold compress 0.9% (n = 1) Child’s mouth 1.7% (n = 4) Adult mouth 1.7% (n = 4)	Liquid medium 94.7% (n = 130) Cold compress 1.8% (n = 2) Adult mouth 1.7% (n = 5)
Statistical significance	*p* < 0.001 *** Cramer’s V 0.320		*p* = 0.509 Cramer’s V 0.100	
**Medium type** (What type of medium do you consider most appropriate?)	Fresh water 12.3% (n = 21) Milk 34.6% (n = 58) Alcohol 3.1% (n = 5) Serum 33.1% (n = 55) Ice water 2.3% (n = 4) Antiseptic solution 14.6% (n = 24)	Fresh water 22% (n = 30) Milk 13.6% (n = 19) Serum 42.0% (n = 58) Ice water 6.6% (n = 9) Antiseptic solution 15.8% (n = 21)	Fresh water 1.7% (n = 3) Milk 96.6% (n = 161) Serum 1.7% (n = 3)	Fresh water 3.5% (n = 5) Milk 94.3% (n = 129) Serum 2.2% (n = 3)
Statistical significance	*p* < 0.001 *** Cramer’s V 0.283		*p* = 0.500 Cramer’s V 0.078	
**Derivation** (Which professional do you consider to be your choice?)	Pediatrician 1.5% (n = 2) Dentist 86.2% (n = 144) Maxillofacial 12.3% (n = 21)	Pediatrician 4.5% (n = 6) Dentist 76.4% (n = 105) Family doctor 1.8% (n = 2) Maxillofacial 17.3% (n = 24)	Dentist 97.4% (n = 163) Maxillofacial 2.6% (n = 4)	Dentist 97.4% (n = 133) Maxillofacial 2.6% (n = 4)
Statistical significance	*p* = 0.116 Cramer’s V 0.157		*p* = 0.983 Cramer’s V 0.105	
**Score mean ± sd** **IC 95%**	5.68 ± 1.86	5.35 ± 1.96	10.37 ± 0.95	10.29 ± 1.01
Statistical significance	*p* = 0.136		*p* = 0.593	

Notes: IC 95% = 95% confidence interval; n = Absolute frequency; Sec: Seconds; SD = standard deviation. Effect size = Cohen’s d = 0.2: Small effect size; 0.5: Moderate effect size; 0.8: Large effect size; Cramer’s V = 0–0.1: Very weak association; 0.1–0.3: Weak association; 0.3–0.5: Moderate association; 0.5–1: Strong association. Statistical significance = * *p* < 0.05, *** *p* < 0.001.

## Data Availability

The data can be provided after contacting the corresponding author.

## Supplementary Materials

The following supporting information can be downloaded at: https://www.mdpi.com/article/10.3390/nursrep14040275/s1, Table S1: MINORS tool (Methodological Index for Non-Randomized Studies); Table S2: Dental trauma knowledge quiz.

## Public Involvement Statement

No public involvement in any aspect of this research.

## Guidelines and Standards Statement

This manuscript was drafted against the STROBE (the full name of reporting guidelines and citation). A complete list of reporting guidelines can be accessed via the equator network: https://www.equator-network.org/ (accessed on 10 February 2024).

## References

[1] Petti S., Andreasen J.O., Glendor U., Andersson L. (2018). The fifth most prevalent disease is being neglected by public health organisations. Lancet Glob. Health.

[2] Karayilmaz H., Kirzioglu Z., Gungor O.E. (2013). Aetiology, treatment patterns and long-term outcomes of tooth avulsion in children and adolescents. Pak. J. Med. Sci..

[3] Petersson E.E., Andersson L., Sörensen S. (1997). Traumatic oral vs non-oral injuries. Swed. Dent. J..

[4] Lembacher S., Schneider S., Lettner S., Bekes K. (2022). Prevalence and Patterns of Traumatic Dental Injuries in the Permanent Dentition: A Three-Year Retrospective Overview Study at the University Dental Clinic of Vienna. Int. J. Environ. Res. Public Health.

[5] de Lima L.G.H., dos Santos C.S., Rocha J.S., Tanaka O., Rosa E.A.R., Gasparello G.G. (2024). Comparative analysis of dental trauma in contact and non-contact sports: A systematic review. Dent. Traumatol..

[6] Faus-Damiá M., Alegre-Domingo T., Faus-Matoses I., Faus-Matoses V., Faus-Llácer V.J. (2011). Traumatic dental injur ies among schoolchildren in Valencia, Spain. Med. Oral Patol. Oral Cir. Bucal.

[7] Mendoza-Mendoza A., Iglesias-Linares A., Yañez-Vico R.M., Abalos-Labruzzi C. (2015). Prevalence and complications of trauma to the primary dentition in a subpopulation of Spanish children in southern Europe. Dent. Traumatol..

[8] Marasca B., Ndokaj A., Duś-Ilnicka I., Nisii A., Marasca R., Bossù M., Ottolenghi L., Polimeni A. (2022). Management of transverse root fractures in dental trauma. Dent. Med. Probl..

[9] Petti S., Glendor U., Andersson L. (2018). World traumatic dental injury prevalence and incidence, a meta-analysis—One billion living people have had traumatic dental injuries. Dent. Traumatol..

[10] Andersson L. (2013). Epidemiology of traumatic dental injuries. Pediatr. Dent..

[11] Traebert J., Peres M.A., Blank V., Böell R., Pietruza J.A. (2003). Prevalence of traumatic dental injury and associated factors among 12-year-old school children in Florianópolis, Brazil. Dent. Traumatol..

[12] Marcenes W., Zabot N.E., Traebert J. (2001). Socio-economic correlates of traumatic injuries to the permanent incisors in schoolchildren aged 12 years in Blumenau, Brazil. Dent. Traumatol..

[13] Magno M.B., Nadelman P., Leite K., Ferreira D.M., Pithon M.M., Maia L.C. (2020). Associations and risk factors for dental trauma: A systematic review of systematic reviews. Community Dent. Oral Epidemiol..

[14] Veras S.R.A., Bem J.S.P., de Almeida E.C.B., Lins C.C.D.S.A. (2017). Dental splints: Types and time of immobilization post tooth avulsion. J. Istanb. Univ. Fac. Dent..

[15] Zaleckiene V., Peciuliene V., Brukiene V., Drukteinis S. (2014). Traumatic dental injuries: Etiology, prevalence and possible outcomes. Stomatologija.

[16] Alotaibi S., Haftel A., Wagner N.D. (2024). Avulsed Tooth. [Updated 6 March 2023]. StatPearls [Internet].

[17] Kalsi H.K., Burns B. (2023). Prevention of Dental trauma. Prim. Dent. J..

[18] Arraj G.P., Rossi-Fedele G., Doğramacı E.J. (2019). The association of overjet size and traumatic dental injuries—A systematic review and meta-analysis. Dent. Traumatol..

[19] Tewari N., Mathur V.P. (2020). Smartphones: The new risk factor for traumatic dental and facial injuries in children. Dent. Traumatol..

[20] Ocak Y., Cicek O., Ozkalayci N., Erener H. (2023). Investigation of the Relationship between Sagittal Skeletal Nasal Profile Morphology and Malocclusions: A Lateral Cephalometric Film Study. Diagnostics.

[21] Azami-Aghdash S., Ebadifard Azar F., Pournaghi Azar F., Rezapour A., Moradi-Joo M., Moosavi A., Ghertasi Oskouei S. (2015). Prevalence, etiology, and types of dental trauma in children and adolescents: Systematic review and meta-analysis. Med. J. Islam. Repub. Iran.

[22] Petrovic B., Marković D., Peric T., Blagojevic D. (2010). Factors related to treatment and outcomes of avulsed teeth. Dent. Traumatol..

[23] Glendor U., Marcenes W., Andreasen J.O., Andreasen J.O., Andreasen F.M., Andersson L. (2007). Classification, epidemiology and etiology. Traumatic Injuries to the Teeth.

[24] Abu-Dawoud M., Al-Enezi B., Andersson L. (2007). Knowledge of emergency management of avulsed teeth among young physicians and dentists. Dent. Traumatol..

[25] Shashikiran N.D., Reddy V.V., Nagaveni N.B. (2006). Knowledge and attitude of 2,000 parents (urban and rural—1,000 each) with regard to avulsed permanent incisors and their emergency management, in and around Davangere. J. Indian Soc. Pedod. Prev. Dent..

[26] Fouad A.F., Abbott P.V., Tsilingaridis G., Cohenca N., Lauridsen E., Bourguignon C., O’Connell A., Flores M.T., Day P.F., Hicks L. (2020). International Association of Dental Traumatology guidelines for the management of traumatic dental injuries: 2. Avulsion of permanent teeth. Dent. Traumatol..

[27] Gonçalves B.M., Dias L.F., Pereira C., Ponte M.X., Filho K.A.C., Bolan M., Cardoso M. (2017). Impact of dental trauma and esthetic impairment on the quality of life of preschool children. Rev. Paul. Pediatr..

[28] Berger T.D., Kenny D.J., Casas M.J., Barrett E.J., Lawrence H.P. (2009). Effects of severe dentoalveolar trauma on the quality-of-life of children and parents. Dent. Traumatol..

[29] Hartmann R.C., Rossetti B.R., Siqueira Pinheiro L., de Figueiredo J.A., Rossi-Fedele G., Gomes M.S., de Borba M.G. (2019). Dentists’ knowledge of dental trauma based on the International Asso-ciation of Dental Traumatology guidelines: A survey in South Brazil. Dent. Traumatol..

[30] Yeng T., O’Sullivan A.J., Shulruf B. (2020). Medical doctors’ knowledge of dental trauma management: A review. Dent. Traumatol..

[31] Antipovienė A., Narbutaitė J., Virtanen J.I. (2021). Traumatic Dental Injuries, Treatment, and Complications in Children and Adolescents: A Register-Based Study, European Journal of Dentistry. https://www.thieme-connect.de/products/ejournals/abstract/10.1055/s-0041-1723066.

[32] Antunes L.S., Debossan P.F., Bohrer L.S., Abreu F.V., Quintanilha L.E., Antunes L.A. (2013). Impact of traumatic dental injury on the quality-of-life of children and adolescents: A case-control study. Acta Odontol. Scand..

[33] Hugar S.M., Suganya M., Kiran K., Vikneshan M., More V.P. (2013). Knowledge and awareness of dental trauma among Indian nurses. Int. Emerg. Nurs..

[34] Suganya M., Vikneshan M., Hiremath A. (2017). Timely management of knocked out teeth—Are the nurses aware?. J. Clin. Nurs..

[35] Khan A., Goyal A., Somaiya V., Rathesh A., Sathiyamoorthy J., Larkin K., Currell S.D., Nimmo A.J. (2020). Knowledge of Australian primary education providers towards dental avulsion injuries: A cross-sectional study. Aust. Dent. J..

[36] Cosme-Silva L., Fernandes L.A., Rosselli E.R., Poi W.R., Martins N.D.S., de Lima D.C. (2018). Tooth Injuries: Knowledge of Parents of Public School Students from the City of Alfenas, Minas Gerais, Brazil. Dent. Traumatol..

[37] Choi D., Badner V.M., Yeroshalmi F., Margulis K.S., Dougherty N.J., Kreiner-Litt G. (2012). Dental trauma management by New York City school nurses. J. Dent. Child.

[38] Coşkun A., Şener A., Şahin O., Ekmekcioğlu C. (2021). Knowledge and Attitudes of Emergency Medicine Physicians and Nurses Regarding Emergency Management of Dentofacial Trauma in Pediatric Patients. Arch. Pediatr..

[39] Baginska J., Rodakowska E., Milewski R., Wilczynska-Borawska M., Kierklo A. (2016). Polish School Nurses’ Knowledge of the First Aid in Tooth Avulsion of Permanent Teeth. BMC Oral Health.

[40] Díaz J., Bustos L., Herrera S., Sepulveda J. (2009). Knowledge of the Management of Paediatric Dental Traumas by Non-Dental Professionals in Emergency Rooms in South Araucanía, Temuco, Chile. Dent. Traumatol..

[41] Trivedy C., Kodate N., Ross A., Al-Rawi H., Jaiganesh T., Harris T., Anderson J.E. (2012). The Attitudes and Awareness of Emergency Department (ED) Physicians Towards the Management of Common Dentofacial Emergencies. Dent. Traumatol..

[42] Gallichan N., Albadri S., Watkins F., Jarad F., Messahel S., Hartshorn S., Gartshore L. (2023). Management of traumatic dental injuries: A survey of paediatric emergency department health professionals. BMJ Paediatr. Open.

[43] Shenoy P., Rao A., Sargod S., Suvarna R., Shabbir A., Mahaveeran S.S., Manu P., Deepanjan M. (2024). Evaluation of Knowledge, Awareness, and Attitude Towards Management of Displaced Tooth from Socket (Avulsion) Amongst Nursing Fraternity in Mangaluru City: A Cross-Sectional Study. J. Health Allied Sci..

[44] Slim K., Nini E., Forestier D., Kwiatkowski F., Panis Y., Chipponi J. (2003). Methodological index for non-randomized studies (minors): Development and validation of a new instrument. ANZ J. Surg..

[45] Faul F., Erdfelder E., Lang A.-G., Buchner A. (2007). G*Power 3: A flexible statistical power analysis program for the social, behav-ioral, and biomedical sciences. Behav. Res. Methods.

[46] Sari S.A., Halabi M.A., Kowash M., Hussein I. (2019). Emergency Management of Traumatic Dental Injuries: Knowledge of Dubai School Nurses. Pesqui. Bras. Em Odontopediatria E Clínica Integr..

[47] Glendor U. (2009). Has the education of professional caregivers and lay people in dental trauma care failed?. Dent. Traumatol..

[48] Pujita C., Nuvvula S., Shilpa G., Nirmala S., Yamini V. (2013). Informative promotional outcome on school teachers’ knowledge about emergency management of dental trauma. J. Conserv. Dent..

[49] Mesgarzadeh A., Shahamfar M., Hefzollesan A. (2009). Evaluating knowledge and attitudes of elementary school teachers on emergency management of traumatic dental injuries: A study in an Iranian urban area. Oral Health Prev. Dent..

[50] McIntyre J.D., Lee J.Y., Trope M., Vann W.F. (2008). Elementary school staff knowledge about dental injuries. Dent. Traumatol..

[51] Bayrak S., Tunc E., Sari E. (2012). Evaluation of elementary school teachers’ knowledge and attitudes about immediate emergency management of traumatic dental injuries. Oral Health Prev. Dent..

[52] Arikan V., Sönmez H. (2012). Knowledge level of primary school teachers regarding traumatic dental injuries and their emergency management before and after receiving an informative leaflet. Dent. Traumatol..

[53] Al-Asfour A., Andersson L., Al-Jame Q. (2008). School teachers’ knowledge of tooth avulsion and dental first aid before and after receiving information about avulsed teeth and replantation. Dent. Traumatol..

[54] Kane A.W., Babacar T., Moustapha D., Malick M., Mouhamed S., Yves B. (2011). Attitude and knowledge of primary school teachers of initial management of dental trauma. J. Oral Health Res..

[55] Udoye C.I., Jafarzadeh H., Abbott P.V. (2012). Transport Media for Avulsed Teeth: A Review. Aust. Endod. J..

[56] Al Sari S., Kowash M., Hussein I., Al-Halabi M. (2019). An Educational Initiative for Dubai School Nurses and Physical Education Teachers on the Management of Traumatic Dental Injuries. J. Sch. Nurs..

[57] Kahabuka F.K., Willemsen W., van’t Hof M., Burgersdijk R. (2001). The Effect of a Single Educational Input Given to School Teachers on Patient’s Correct Handling After Dental Trauma. SADJ J. S. Afr. Dent. Assoc..

[58] Al-Musawi A., Al-Sane M., Andersson L. (2017). Smartphone App as an Aid in the Emergency Management of Avulsed Teeth. Dent. Traumatol..

[59] Fittler M., Fittler A., Dergez T., Radácsi A., Katona K., Sándor B., Szántó I. (2024). Knowledge and management of traumatic dental injuries among schoolteachers in Hungary: A cross-sectional study with educational intervention. Eur. Arch. Paediatr. Dent..

[60] Tewari N., Jonna I., Mathur V.P., Goel S., Ritwik P., Rahul M., Haldar P., Bansal K., Pandey R.M. (2021). Global Status of Knowledge for the Prevention and Emergency Management of Traumatic Dental Injuries Among Non-Dental Healthcare Professionals: A Systematic Review and Meta-Analysis. Injury.

[61] Modha B. (2021). Collaborative leadership with a focus on stakeholder identification and involvement and ethical leadership: A dental perspective. Br. Dent. J..

[62] Ministry of Universities (2023). Organic Law 4/2023, of June 28, of the University System (LOSU). Off. State Gaz..

