# Supplementary material for: Enhancing Nursing Competencies: An Assessment of Knowledge and Attitudes Toward Dental Trauma Management Among Nursing Students—An Interventional Study
Source: Nurs Rep. 2024 Nov 29;14(4):3765–78. doi: 10.3390/nursrep14040275 (PMC11677559; doi:10.3390/nursrep14040275)
Supplement: Supplementary file 1 [file nursrep-14-00275-s001.zip › nursrep-3246159-figures.pdf]

TABLA S2: Dental trauma knowledge quiz

1.**SEX:**	
	Male
	Female
2.**AGE** (YEARS)	
3.**WHAT NURSING DEGREE COURSE ARE YOU IN?**	
	Third
	Fourth
4.**HAVE YOU PREVIOUSLY WORKED IN THE DENTAL FIELD?**	
	Yes
	Not
5.**IF YES, SPECIFY WHAT POSITION YOU HELD:**	
6.**HAVE YOU TRAINED PREVIOUSLY IN DENTAL TRAUMA?**	
	Yes
	Not
7.**HAVE YOU EVER WITNESSED A DENTAL TRAUMA ACCIDENT?**	
	Yes
	Not
8.**DO YOU THINK YOU HAVE THE KNOWLEDGE NECESSARY TO MANAGE A TRAUMATIC DENTAL INJURY?**	
	Yes
	Not
9.**DO YOU THINK YOU NEED MORE KNOWLEDGE/TRAINING REGARDING DENTAL TRAUMATOLOGY?**	
	Yes
	Not
10.**ARE YOU ABLE TO DISTINGUISH BETWEEN A TEMPORARY TOOTH AND A PERMANENT ONE?**	
	Yes
	Not
11.**THE TEETH SEEN IN THE IMAGE, ARE TEMPORARY TEETH OR PERMANENT TEETH?**	
	Temporary
	Permanent
12.**WOULD YOU KEEP THE FRAGMENTS?**	
	Yes
	Not
13. **WHAT TYPE OF MEDIUM WOULD YOU PRESERVE IT ON?**	
	Wet
	Dry

14. **DO YOU THINK IT IS POSSIBLE TO RELOCATE THE AVULSED TOOTH?**	
	Yes
	Not
15. **WOULD YOU BE ABLE TO REIMPLANT IT?**	
	Yes
	Not
16. **IN WHAT TIME LIMIT DO YOU THINK IT SHOULD BE RELOCATED?**	
	Any time
	First 5 hours
	After two hours to rehydrate
	As soon as possible, without limit
	In the first 24 hours
17. **GIVEN THAT THE TOOTH IS DIRTY, HOW WOULD YOU HANDLE IT?**	
	I would replace without washing
	Wash with saliva
	Wash 10 sec
	Wash with milk
	Wash with soap
	Rub with brush
18. **WHAT TYPE OF TRANSPORTATION DO YOU CONSIDER MOST APPROPRIATE?**	
	Liquid medium
	Cold compress
	Child's mouth
	Adult mouth
	Wrapped in paper
	Plastic container
19. **WHAT TYPE OF MEDIUM DO YOU CONSIDER MOST APPROPRIATE?**	
	Fresh water
	Fresh milk
	Alcohol
	Serum
	Ice water
	Antiseptic solution
20.**WHICH PROFESSIONAL DO YOU CONSIDER TO BE YOUR CHOICE?**	
	Pediatrician
	Dentist
	Family doctor
	Maxillofacial
