# Supplementary material for: Enhancing Nursing Competencies: An Assessment of Knowledge and Attitudes Toward Dental Trauma Management Among Nursing Students—An Interventional Study
Source: Nurs Rep. 2024 Nov 29;14(4):3765–78. doi: 10.3390/nursrep14040275 (PMC11677559; doi:10.3390/nursrep14040275)
Supplement: Supplementary file 1 [file nursrep-14-00275-s001.zip › nursrep-3246159-supplementary-3.pdf]

Table S1: MINORS tool (Methodological Index for Non-Randomized Studies)

	Score
**1. A clearly stated aim**	2
**2. Inclusion of consecutive patients**	2
**3. Prospective collection of data**	2
**4. Endpoints appropriate to the aim of the study**	2
**5. Unbiased assessment of the study endpoint**	1
**6.Follow-up period appropriate to the aim of the study**	1
**7. Loss to follow up less than 5%**	2
**8. Prospective calculation of study size**	2
Additional criteria in the case of comparative studies.	
**9. An adequate control group**	Not applicable
**10 Contemporary groups**	Not applicable
**11. Baseline equivalence of groups:**	Not applicable
**12. Adequate statistical analyses**	Not applicable
**Total, score**	14

0 points: if not reported; 1 point: if it is reported inadequately; 2 points: if it is reported appropriately.
